# Inflammatory bowel disease and rheumatoid arthritis share a common genetic structure

**DOI:** 10.3389/fimmu.2024.1359857

**Published:** 2024-06-13

**Authors:** Guoling Cao, Qinghua Luo, Yunxiang Wu, Guanghua Chen

**Affiliations:** ^1^ Department of Anorectal Surgery, The People’s Hospital of Cangnan, Wenzhou, China; ^2^ Clinical Medical College, Jiangxi University of Chinese Medicine, Nanchang, China; ^3^ Department of Anorectal Surgery, Affiliated Hospital of Jiangxi University of Chinese Medicine, Nanchang, China

**Keywords:** rheumatoid arthritis, inflammatory bowel disease, genetic risk loci, genetic structure, GWAS, gut joint axis

## Abstract

**Background:**

The comorbidity rate of inflammatory bowel disease (IBD) and rheumatoid arthritis (RA) is high; nevertheless, the reasons behind this high rate remain unclear. Their similar genetic makeup probably contributes to this comorbidity.

**Methods:**

Based on data obtained from the genome-wide association study of IBD and RA, we first assessed an overall genetic association by performing the linkage disequilibrium score regression (LDSC) analysis. Further, a local correlation analysis was performed by estimating the heritability in summary statistics. Next, the causality between the two diseases was analyzed by two-sample Mendelian randomization (MR). A genetic overlap was analyzed by the conditional/conjoint false discovery rate (cond/conjFDR) method.LDSC with specific expression of gene analysis was performed to identify related tissues between the two diseases. Finally, GWAS multi-trait analysis (MTAG) was also carried out.

**Results:**

IBD and RA are correlated at the genomic level, both overall and locally. The MR results suggested that IBD induced RA. We identified 20 shared loci between IBD and RA on the basis of a conjFDR of <0.01. Additionally, we identified two tissues, namely spleen and small intestine terminal ileum, which were commonly associated with both IBD and RA.

**Conclusion:**

Herein, we proved the presence of a polygenic overlap between the genetic makeup of IBD and RA and provided new insights into the genetic architecture and mechanisms underlying the high comorbidity between these two diseases.

## 1 Introduction

Inflammatory bowel disease (IBD) is a common, chronic, nonspecific, immunologic clinical disease of the gastrointestinal tract (1). Clinically, the condition is generally categorized as either Crohn’s disease (CD) or ulcerative colitis (UC). There are distinct differences between UC and CD. UC typically presents with continuous mucosal inflammatory lesions extending from the rectum to the proximal colon, whereas CD can affect any segment of the gastrointestinal tract and is characterized by transmural inflammation and cobblestone-like changes (2). Patients with IBD commonly experience abdominal pain, increased stool frequency, weight loss, and multiple extra-intestinal symptoms (3, 4). Over the last few decades, the number of people suffering from the disease has increased significantly in Western countries (5). Almost 60,000 cases of IBD were reported worldwide in 2017 (6). Clinically, IBD is associated with several immune-mediated diseases including rheumatoid arthritis (RA) (7). RA is the most common chronic autoimmune disease of the synovial joints (8) and is characterized by intra-articular manifestations, such as the swelling of and deformity in the joints, and extra-articular manifestations, including rheumatoid nodules, vasculitis, pleural effusions, and accelerated atherosclerosis (9). Both IBD and RA are multifactorial diseases influenced by environmental factors. Risk factors for RA include exposure to tobacco smoke, occupational dust, high sodium diet, and excessive red meat consumption (10). Microorganisms such as mycoplasma, Epstein-Barr virus (EBV), porphyromonas, Proteus mirabilis, and Mycobacterium avium paratuberculosis (MAP) have been implicated in the pathogenesis of RA (11, 12). Similarly, IBD is affected by smoking, psychological stressors,pollutants in the air and water,dietary habits,and bacterial and viral infections (13–15).

IBD and RA analysis at the genetic level may reveal potential associations between them. TNF-like ligand 1A(TL1A), a common genetic risk locus between IBD and RA, competitively binds to death receptor 3(DR3) or decoy receptor 3(DcR3), stimulates downstream signaling pathways, and affects the regulation of effector cell proliferation, activation, and apoptosis and the production of cytokines and chemokines, which eventually affect IBD and RA progression (16, 17). Additionally, polymorphisms in the interferon regulatory factor 5(IRF5) and TNF receptor superfamily member 14(TNFRSF14) motifs can predispose the susceptibility of IBD and RA (18–21). Presently, detailed studies on mining shared genetic risk loci between IBD and RA are lacking; therefore, herein, we focused on identifying risk loci for these comorbidities.

Existing genome-wide association studies (GWAS) are developing rapidly over time, and GWAS on IBD and RA can help elucidate the underlying genetic mechanisms. While conducting common genetic structure analysis of complex diseases, it is not advisable to simply take the GWAS sites shared by two diseases. The reason is their genetic risk architecture is generally dependent on common genetic variants having small effect sizes, which can easily lead to false-negative results (22). Nevertheless, this error can be avoided by employing some novel genetic statistics methods and the genetic architecture of both diseases can be effectively studied (23–25). To better understand the common genetic structure between IBD and RA, we employed such methods.

In the present study, we first analyzed overall and local genetic correlations; a linkage disequilibrium score regression (LDSC) analysis was performed for the former (26), whereas estimated heritability in summary statistics (HESS) was performed for the latter (27). Subsequently, Mendelian randomization (MR) was used for analyzing causal correlations (28). Additionally, we investigated the potential genetic correlation between IBD and RA regarding a multigene overlap by following the conditional/conjoint false discovery rate (cond/conjFDR) method, which helped identify concordant genetic risk variant loci (29). The LDSC with specific expression of gene (LDSC–SEG) approach was used to identify tissues related to the two diseases (30). Finally, GWAS multitrait analysis (MTAG) was performed for the two diseases (31). We hope that the present study will help better understand shared genetic structures and molecular mechanisms between IBD and RA.

## 2 Methods and materials

### 2.1 GWAS data

**Figure 1:**
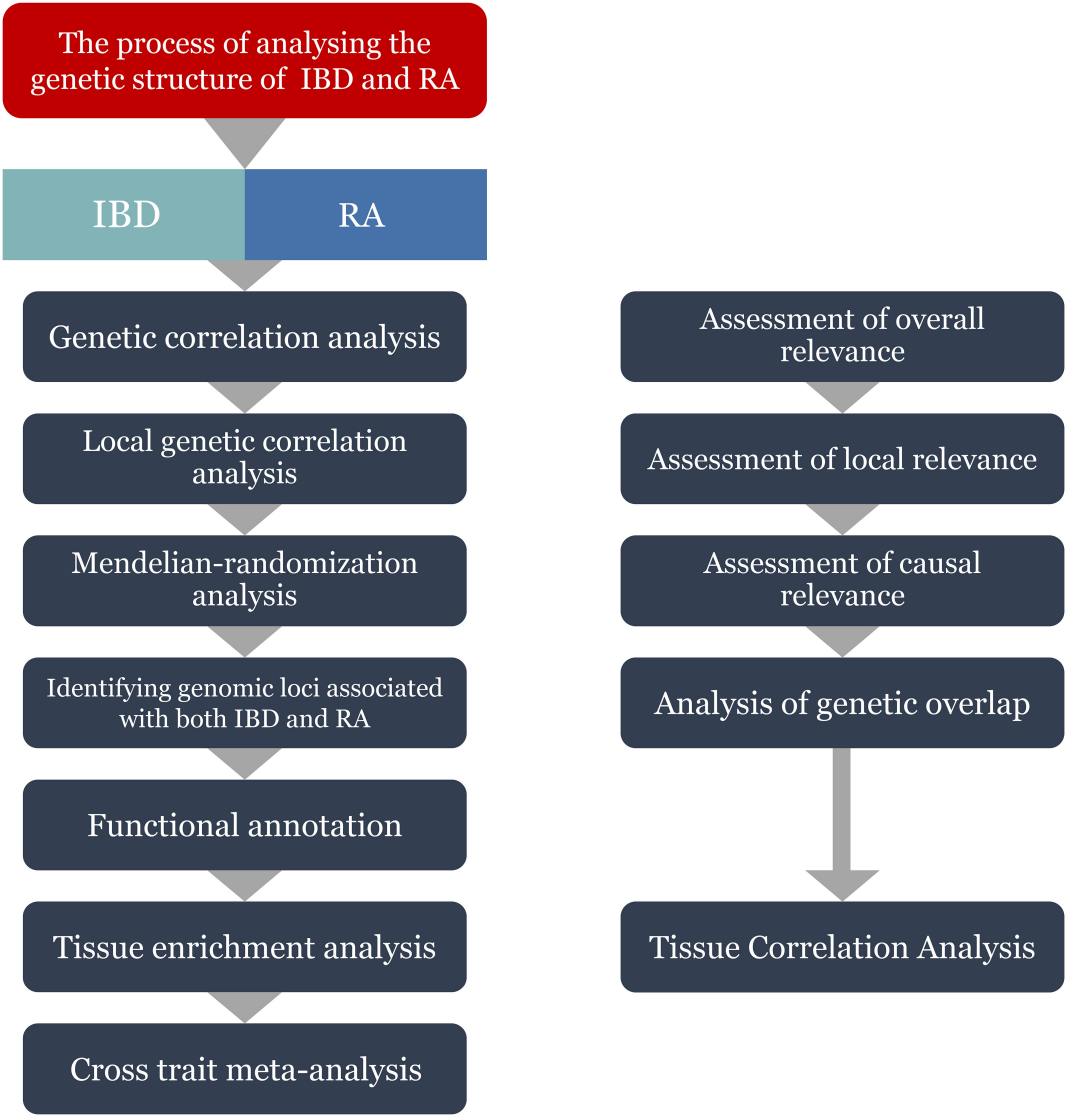
Flowchart of the study. IBD, inflammatory bowel disease; RA, Rheumatoid arthritis.

Data on IBD was collected from the IEU GWAS database (https://gwas.mrcieu.ac.uk/), and GWAS summary statistics for IBD (ID: ebi-a-GCST004131), CD (ID: ebi-a-GCST004132) and UC (ID: ebi-a-GCST004133) were obtained from the European Bioinformatics Institute (EBI) database (32). Among them, IBD data includes a total of 59957 samples, including 25042 patients and 34915 healthy controls; The data of CD includes a total of 40266 samples, including 12194 patients and 28072 healthy controls; UC’s data includes a total of 45975 samples, including 12366 patients and 33609 healthy controls. The latest summary-level statistics for RA were obtained from the FinnGen GWAS results (https://r10.finngen.fi/) (33), which comprised a total of 12,555 patients with RA and 240,862 controls. All the participants were of European ancestry. We also selected the RA data (ID: ukb-b-9125) from the IEU database for validation.The flow chart of the present study is presented in **Figure 1**.

### 2.2 Genetic correlation analysis

LDSC (version 1.0.1) calculates the genetic correlation between two shapes to show the average degree of the sharing of genetic effects between the pairs of traits (30). Genetic correlation (rg), the most important objective of the present study, ranges from −1 to 1, with the “−” sign denoting a negative correlation and the “+” sign denoting a positive correlation. Per the LDSC manual, the first step is to convert all GWAS summary statistics into the LDSC format with the help of the default parameters of munge_sumstats.py provided in the package. The second step is to calculate genetic correlations using the -rg, -ref-ld-chr, and -w-ld-chr parameters in ldsc.py. The precalculated LD scores passed to the -ref-ld-chr and -w-ld-chr parameters can be downloaded from https://alkesgroup.broadinstitute.org/LDSCORE/. Moreover, information on European pedigrees from the 1000 Genomes Project (34), which was consistent with the European pedigrees of the GWAS samples, was used as a reference panel for chain imbalance.

### 2.3 Local genetic correlation analysis

In the local genetic correlation analysis, we investigated 1703 gene segments that were delineated and that should be independent of prespecified LDs. HESS, a novel computational tool, calculates local SNP heritability and measures the degree of similarity between the pairs of traits driven by genetic variation (27). However, the HESS results should be corrected using the Bonferroni method, for which a correlation was considered statistically significant at P < 0.05/1703 = 2.94E-05.

### 2.4 MR analysis

**Figure 2:**
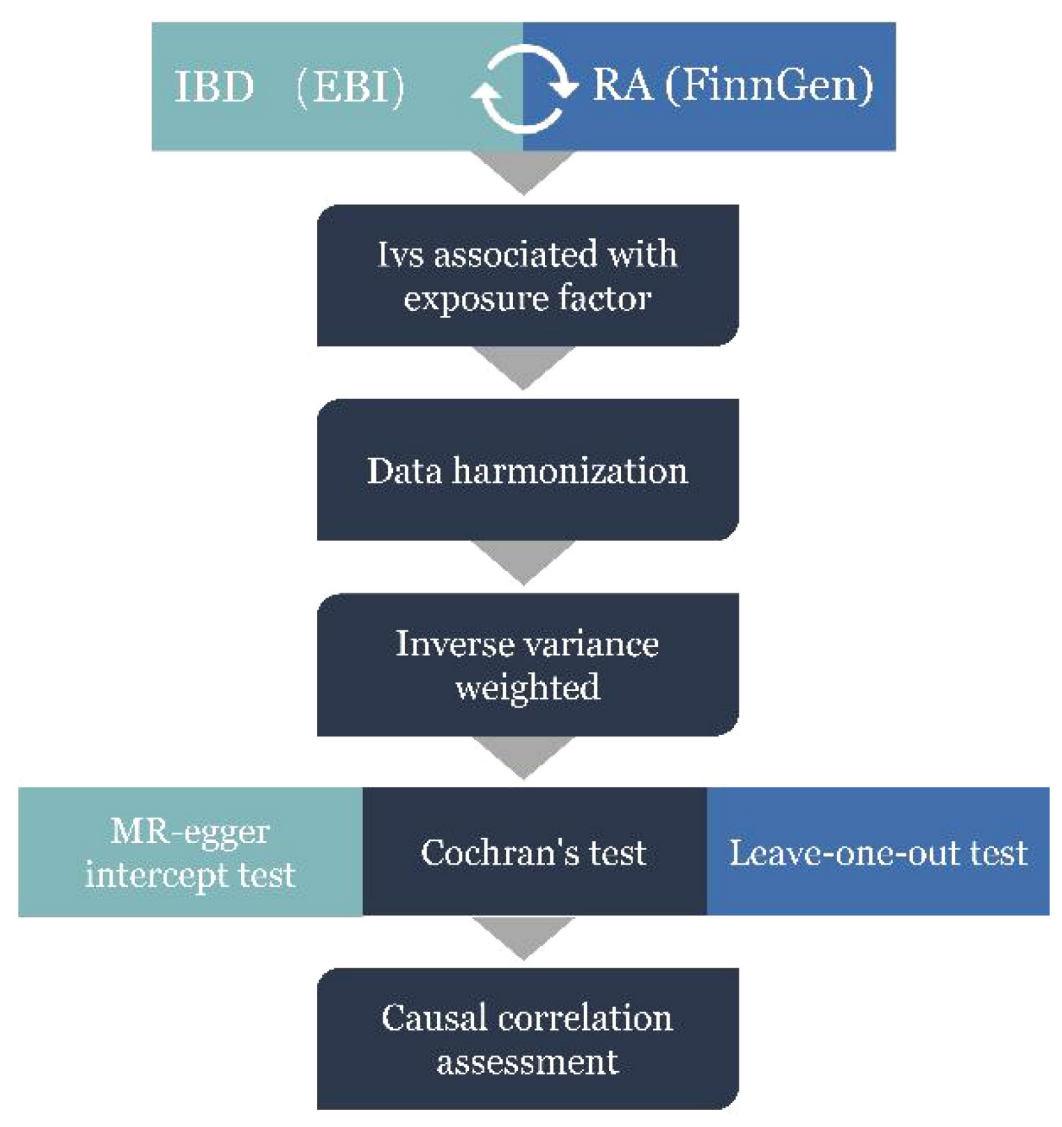
MR Analysis process and acquisition of valid instrumental variables (IV) steps. IBD, inflammatory bowel disease; RA, Rheumatoid arthritis.

The specific procedure followed for the MR analysis is presented in **Figure 2**. First, according to the correlation assumption of MR, we set the P-value to be less than 5 × 10−8 to obtain single nucleotide polymorphisms (SNPs) that are closely related to exposure. Second, LD was excluded by setting the r2 value to 0. 001 and kilobase pairs (kb) to 10 000 (35). Additionally, we manually input all exposure-related SNPs into Phenoscanner (http://www.phenoscanner.medschl.cam.ac.uk/) to check for any confounding variables related to outcomes. If confounding variables were present, the corresponding SNPs were deleted to satisfy the assumption that IVs is independent of confounding factors and outcomes.

Pleiotropy, heterogeneity, and sensitivity are important tools for the quality control of the MR analysis results. Based on the results of two polyvalence assessments, MR-Egger regression (36) and MR-PRESSO test (37), we identified abnormal SNPs, which were rejected. The results of inverse variance weighted (IVW) and MR Egger methods, including Cochran’s Q statistic and the corresponding P-value, where the P-value is the weighing of the presence and absence of heterogeneity (P < 0.05 denoting no heterogeneity), showed heterogeneity. The leave-one-out method eliminates each SNP in turn to calculate the combined effect of the remaining SNPs in order to determine whether the SNP exerts an unfavorable main effect on the overall causal relationship (38). Additionally, we analyzed the eligibility of the included SNPs by calculating the F-value (F = β2/SE2), where β is the effect value of the allele, and SE is the standard error (39). An F-value greater than 10 suggested the eligibility and absence of weak instrumental variables and vice versa (40).

TwosampleMR was used for the MR analysis, and IVW was selected as the stochastic model; this is the most critical method for evaluating analysis results. IVW evaluates the average causal effect between two traits, and the evaluation includes calculating the Wald ratio estimates for each SNP (41). The entire MR analysis is bidirectional.

### 2.5 Identifying shared genomic loci between IBD and RA

The empirical Bayesian statistical framework of condFDR and conjFDR analyses was used to identify shared genetic risk loci for IBD and RA (42). First, conditional quantile–quantile (Q–Q) plots were generated, which showed overlapping SNP associations among different traits. The conditional Q–Q plot analysis sets SNPs against secondary traits as a reference and evaluates all SNPs against the primary traits. Second, condFDR was used as a reference to reorder the test statistics of the primary trait (e.g., IBD) on the basis of the strength of the association with the secondary trait (e.g., RA) to enhance the feasibility of SNP identification (29). Next, the primary and secondary traits were inverted to perform a reverse condFDR analysis. The loci of the primary and secondary traits were considered significant at condFDR ≤ 0.01. Finally, the maximum value of condFDR was set as the value of conjFDR to ensure that the FDR associated with the two traits was more accurate. The entire analysis was performed by directly eliminating SNPs around the major histocompatibility complex expansion region, which improved the accuracy of the FDR estimates (43). The detailed procedure for the conjFDR analysis is available at https://github.com/precimed/pleiofdr.

### 2.6 Functional annotation

FUMA (44), an online analysis website (available at https://fuma.ctglab.nl/), allows for the positional mapping and functional annotation of new, shared, and specific locus. We considered candidate SNPs with conjFDR < 0.05 and LD r2 < 0.6 with each other as independent significant SNPs. Among them, those SNPs with LD r2 < 0.1 were designated as lead SNPs. All the candidate SNPs in LD r2 ≥ 0.6 with a lead SNP demarcated the boundaries of a genomic locus. We merged loci separated by less than 250 kb. Therefore, any candidate SNP located within the boundaries of a genomic locus was defined to belong to a single independent genomic locus. We also employed the Combined Annotation Dependent Depletion (CADD), a framework that objectively integrates diverse annotations into a unified quantitative score, which is pivotal in evaluating functional, deleterious, and pathogenic genetic variations. A CADD score exceeding 12.37 signifies harmfulness (45).

We investigated pathway enrichment for the mapped genes. Gene ontology (GO) annotation and Kyoto Encyclopedia of Genes and Genomes (KEGG) pathway enrichment analyses were performed to identify common trends related to the function of differentially expressed genes (46), which was achieved by performing GO annotations of genes from the R package org.Hs.eg.db (version 3.1.0). The genes were mapped to the background set, and the enrichment analysis was performed using the R package clusterProfiler (version 3.14.3). The minimum gene set included 5 entities and the maximum gene set included 5000 entities, and the P-value and FDR value were set to less than 0.05 and less than 0.1, respectively. For this, the enrichment analysis tool of the Sangerbox platform (http://vip.sangerbox.com/) was used (47).

### 2.7 Tissue enrichment analysis

The LDSC–SEG analysis was performed by considering the tissue gene expression data as a reference and combining the GWAS data of both IBD and RA to identify tissues associated with IBD and RA (30). In this analysis, the t-statistic of each gene expressed in different tissues was calculated, the t-statistic scores of all genes were ranked in ascending order, and the top 10% of the genes were considered as the gene set corresponding to the lesion tissue. Further, the gene sets were divided into strong and weak specific expression gene sets per their intensity. In each gene set, a 100-kb window was set between the front and back of the transcribed region of each gene in order to establish the annotation information of the tissue genome. Finally, GWAS summary statistics were performed to assess the role of focused genome annotation in trait heritability. GTEx (v8) offers insights into 53 distinct tissue types, encompassing data on SNP mutations linked to quantitative traits of gene expression across various tissues (48). S-LDSC provides preprocessed reference comparison panels for 53 tissues of the GTEx project in this version, which allows researchers to more conveniently evaluate the correlation between tissues and traits. We downloaded and used pre-calculated GTEx project gene expression data as genomic annotations for each tissue to reveal the potential tissue origins of the comorbidity inheritance between IBD and RA. The detailed procedure of the LDSC–SEG analysis is available at https://github.com/bulik/ldsc/wiki/Cell-type-specific-analyse.

### 2.8 Cross-trait meta-analysis

Utilizing Python 3.11.5, we conducted a GWAS multi-trait analysis (MTAG) between IBD and RA to ascertain the risk SNPs significantly associated with both traits (31). MTAG facilitates the joint analysis of GWAS statistical summaries for different traits without potential sample overlap between studies. It is predicated on the establishment of a variance-covariance matrix with shared effect sizes across traits (31). The GWAS derived from MTAG analysis between the two traits was submitted to the FUMA (44) online analysis platform for identification of the associated genetic risk loci.

## 3 Results

### 3.1 Genetic correlation

The LDSC analysis results showed 2380 SNPs that were significantly correlated to IBD and 773 SNPs that were significantly correlated to RA.2076 and 1042 on the CD and UC side, respectively. Excluding the intercept, the probability of inheritance for IBD was 31.3%, whereas that for RA was 2.73%, and a positive correlation was observed between the two (rg = 0.1279, P = 0.0191).The correlation of RA with CD (rg = 0.1242, P= 0.0347) was also positive, but there was no significant association in terms of UC (rg = 0.1006, P= 0.0756). The results of the validation were consistent with the above performance (**Table 1**).

**Table 1:** Genetic correlation of RA and IBD(including CD and UC).

Trait1	Trait2	H2(trait1)	H2(trait2)	Rg	Se	P
IBD	RA	0.3130	0.0273	0.1279	0.0546	0.0191
CD	RA	0.4457	0.0273	0.1242	0.0588	0.0347
UC	RA	0.2387	0.0273	0.1006	0.0566	0.0756
IBD	ukb-b-9125	0.3130	0.0045	0.2468	0.0921	0.0074
CD	ukb-b-9125	0.4457	0.0045	0.2450	0.0865	0.0046
UC	ukb-b-9125	0.2387	0.0045	0.1707	0.1092	0.1181

H2: Represents the observed genetic contribution, the larger the better. Rg: Correlation between two traits, rg ranges from -1 to 1, and the closer the value is to 1 or -1, the stronger the correlation is (plus or minus represents positive and negative correlation). Se: standard error of genetic correlation. **P**: the statistically significant association is defined to be p.

**Figure 3:**
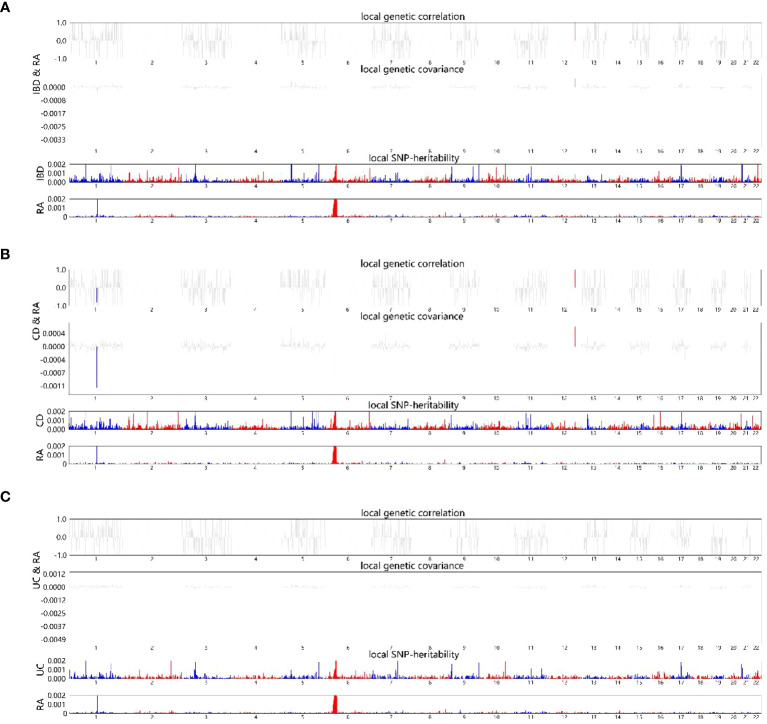
HESS analysis of RA and IBD, CD and UC. The top and middle sections of each subgraph represent local genetic correlations and covariances, respectively, and the colored bars represent loci with significant local genetic correlations and covariances. The bottom portion represents the local snp heritability of an individual trait, and the colored bars represent loci with significant local snp heritability. **(A)** Local genetic correlation between IBD and RA. **(B)** Local genetic correlation between CD and RA. **(C)** Local genetic correlation between UC and RA. RA, Rheumatoid arthritis; IBD, inflammatory bowel disease; CD, Crohn’s disease; UC, ulcerative colitis.

The local genetic correlation mapping analysis indicated a local genetic overlap between IBD and RA, and the specific site was on two chromosomes, 6 and 12. CD aspects are expressed on chromosomes 1 and 12 and UC on chromosome 6 (**Figures 3A-C**). Localized correlations in ukb-b-9125 are enriched on chromosomes 1 and 6 (**Supplementary Figures 1A-C**).

### 3.2 MR

**Figure 4:**
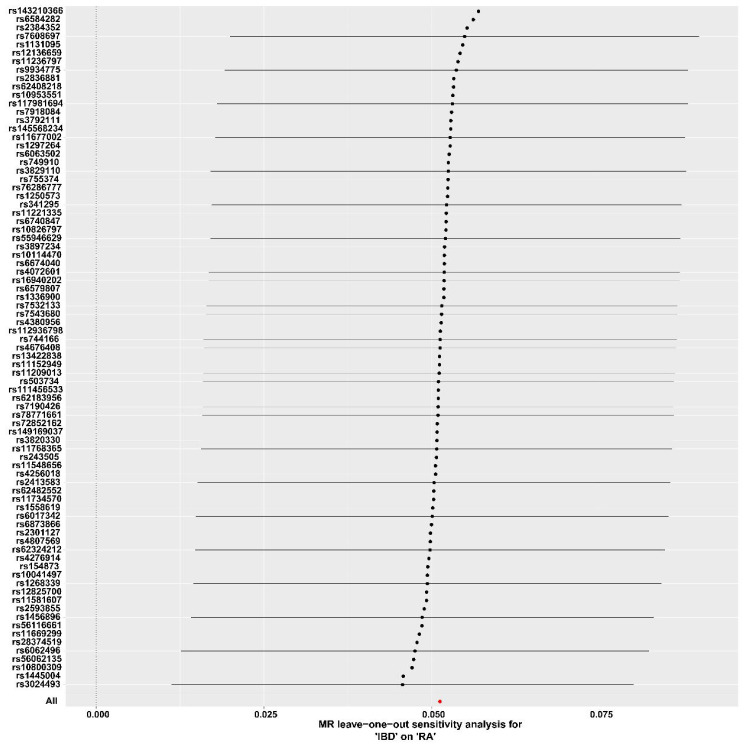
Forest plot for the leave-one-out analysis. IBD, inflammatory bowel disease; RA, Rheumatoid arthritis.

In the MR analysis of IBD on RA orientation, 81 SNPs were studied. The IVW results showed a positive causal effect of IBD on RA risk (P = 0.0039). The leave-one-out analyses showed no potentially affecting SNP-driven causal associations, indicating the reliability of the present results (**Figure 4**). Additionally, MR-PRESSO showed no horizontal multidirectionality (P = 0.3873). The reverse MR analysis showed no significant causal effect of RA on IBD.There was no causal relationship between IBD subtypes and RA (**Supplementary Table 1**). There were too few reverse dependent instrumental variables, and only positive MR was performed for IBD and ukb-b-9125, with results suggesting no causality.(**Supplementary Table 1**). The F-values for all instrumental variables were greater than 10, which suggested the absence of a bias in the weak instrumental variables (**Supplementary Table 1**).

### 3.3 conjFDR analysis to identify shared genomic loci between IBD and RA

**Figure 5:**
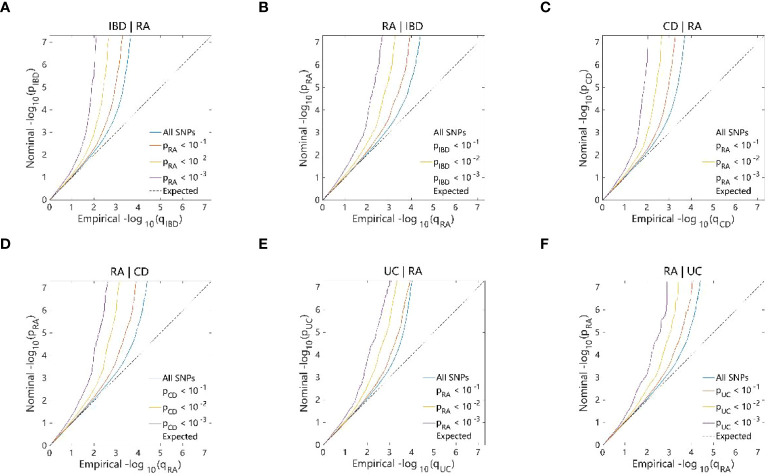
Conditional quantile-quantile plot. The dashed line indicates the expected line under the null hypothesis, and the deflection to the left indicates the degree of pleiotropic enrichment. **(A)** IBD-RA. **(B)** RA-IBD. **(C)** CD-RA. **(D)** RA-CD. **(E)** UC-RA. **(F)** RA-UC. RA, Rheumatoid arthritis; IBD, inflammatory bowel disease; CD, Crohn’s disease; UC, ulcerative colitis.

**Figure 6:**
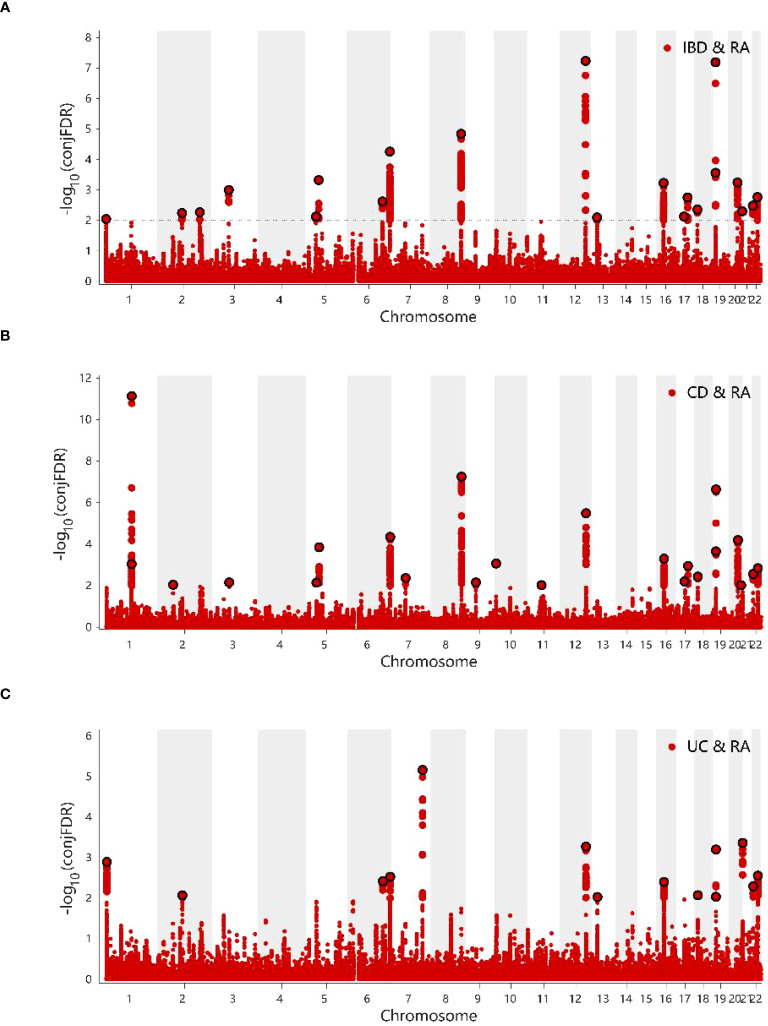
**(A)** ConjFDR Manhattan plot of IBD and RA. **(B)** ConjFDR Manhattan plot of CD and RA. **(C)** ConjFDR Manhattan plot of UC and RA. The shared risk loci between RA and IBD, CD and UC were marked. The statistically significant causality is defined to be conjFDR <0.01. RA, Rheumatoid arthritis; IBD, inflammatory bowel disease; CD, Crohn’s disease; UC, ulcerative colitis.

The conditional Q–Q plots for IBD and RA showed that when the association significance of one disease increased, the other disease significantly shifted to the left from the expected zero line, which suggested the presence of genetic enrichment and a shared genetic background between IBD (including CD and UC) and RA (**Figures 5A-F**). The same conclusion was reached with respect to ukb-b-9125 (**Supplementary Figures 2A-F**). The ConjFDR analysis showed that all genes between the two traits overlapped in a way that the overlapping improved the qualitative confidence and guaranteed that the identified loci belonged to both diseases. At conjFDR < 0.01, there were 20 genetic risk loci shared by IBD GWAS and RA GWAS (**Figure 6A**; **Supplementary Table 2**), of which 17 showed the same direction of effect (**Supplementary Table 2**). For CD and UC, the genetic risk loci were 21 and 13, respectively, of which 16 and 13 showed the same direction of effect (**Figures 6B, C**; **Supplementary Tables 3**, **4**). Ukb-b-9125 had fewer genetically risk loci between ukb-b-9125 and IBD, CD, and UC, corresponding to 1, 3, and 0, respectively (**Supplementary Figures 3A-C**).

### 3.4 Functional annotations

At concFDR < 0.01, a total of 92 mapping genes (**Supplementary Table 5**) and 1575 candidate SNPs were obtained for IBD and RA, which mainly corresponded to ncRNA_intronic (n = 544, 34.8%), intronic (n = 530, 34.0%), and intergenic (n = 327. 20.9%) functions (**Supplementary Table 6**). Among the 20 genetic risk loci, the functional attributes intronic、intergenic、ncRNA_intronic、downstream, and ncRNA_exoni corresponded to 9, 5, 3, 2, and 1 SNPs, respectively (**Supplementary Table 2**). Among these 20 enetic risk loci, the combined annotation-dependent depletion value of the SNP rs353341 exceeded the threshold of 12.37, which indicated that it was deleterious (45). There are 2061 and 1311 candidate SNPs for CD and UC, respectively (**Supplementary Tables 7**, **8**). For mapping genes, there are 103 for CD and 67 for UC (**Supplementary Tables 9**, **10**).

**Figure 7:**
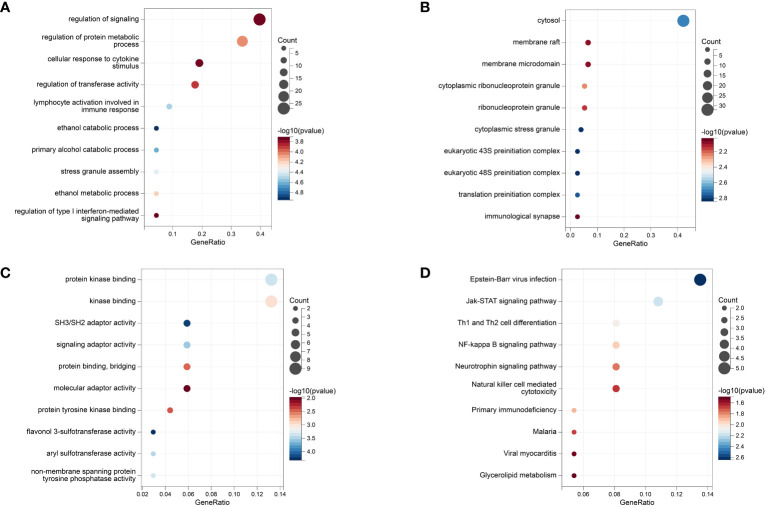
The Results of Enrichment analysis. **(A)** GO enrichment analysis at biological process. **(B)** GO enrichment analysis at molecular function. **(C)** GO enrichment analysis at cell composition. **(D)** KEGG analysis.

The GO annotation and KEGG pathway enrichment analyses were performed on the 92 mapped genes in IBD, and the results showed that in terms of biological processes, the top three aspects of gene enrichment were signaling regulation, protein metabolism regulation, and cellular response to cytokine stimulus (**Figure 7A**). In terms of cellular components, the cytosol, membrane raft, and membrane microdomain were the main directions of gene enrichment (**Figure 7B**). In terms of molecular functions, genes were mainly enriched for protein kinase binding and kinase binding (**Figure 7C**). Conversely, the notable pathways analyzed by KEGG were Epstein–Barr virus (EBV) infection, Janus kinase signal transducer and activator of transcription (JAK–STAT) signaling pathway, and Th1 and Th2 cell differentiation (**Figure 7D**). The results of the enrichment analysis for CD and UC can be seen in **Supplementary Figures 4**, **5**.

### 3.5 Trait-related tissue

**Figure 8:**
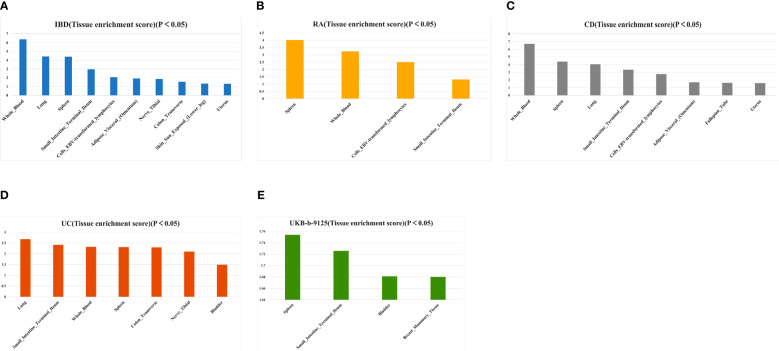
Tissues enrichment results of IBD **(A)**, AS **(B)**, CD **(C)**, UC **(D)**, and ukb-b-9125 **(E)** using gene expression data of 53 tissues from GTEx. RA, Rheumatoid arthritis; IBD, inflammatory bowel disease; CD, Crohn’s disease; UC, ulcerative colitis.

Based on the tissue expression data from GTEx, we performed additional LDSC–SEG analyses to identify disease-associated tissues. At P < 0.05, IBD was associated with the whole blood, lung, spleen, small intestine terminal ileum, EBV-transformed lymphocytes, adipose visceral (omentum), nerve tibial, transverse colon, sun-exposed skin (lower leg), and uterus (**Figure 8A**; **Supplementary Table 11**). Tissues that showed a significant correlation with RA were the spleen, whole blood, EBV-transformed lymphocytes, and small intestine terminal ileum (**Figure 8B**; **Supplementary Table 12**). Crohn’s disease is closely associated with whole blood, spleen, lungs, terminal ileum of the small intestine, EBV-transforming lymphocytes, fatty viscera (omentum), fallopian tubes, and uterus (**Figures 8C**; **Supplementary Table 13**). Tissues significantly associated with UC include the lung, terminal ileum of the small intestine, whole blood, spleen, transverse colon, tibial nerve and bladder (**Figures 8D**; **Supplementary Table 14**). LDSC-SEG results for ukb-b-9125 indicated significant associations with spleen tissue as well as the terminal ileum of the small intestine; also showing significant association with bladder and mammary gland tissue (**Figures 8E**; **Supplementary Table 15**). The spleen and small intestine terminal ileum were common to both diseases, which suggested that IBD and RA might have a common tissue origin. The relevant specific analyses and their results are presented in **Supplementary Tables 11**-**15**.

### 3.6 MTAG

After performing MTAG analysis and Fuma annotation for both IBD and RA, a total of 94 genetic risk loci were identified (**Supplementary Table 16**). The conjfdr and MTAG analysis revealed the intersection of five genes (FOXP1, RP11–95M15.1, RP11–89M16.1, PTPN2, and UBE2L3) (**Supplementary Figure 6A**). For CD, we found 80 genetic risk loci (**Supplementary Table 17**), with the corresponding intersection genes being AC020743.4, RP11–89M16.1, PTPN2, and UBE2L3 (**Supplementary Figure 6B**). Upon analyzing UC, only 49 genetic risk loci were identified (**Supplementary Table 18**), with the intersection genes being RP11–95M15.1 and IRF5 from the two analyses (**Supplementary Figure 6C**).

## 4 Discussion

In the present study, the results of LDSC and HESS suggested a genetic correlation between IBD and RA, at both the overall and local levels. Subsequently, we performed a conjFDR analysis at the SNP level to mine novel genetic variants associated with the two shapes of IBD and RA. Finally, considering tissue genetic data as a reference, we performed an LDSC–SEG analysis to obtain two common tissue sources, namely the spleen and small intestine terminal ileum and provided favorable histologic evidence. The present results showed the genetic association between IBD and RA and provided insights into the possible regulatory functions of the shared genetic factors. Nevertheless, the functions need further investigation.

Several existing cross-sectional observational studies have suggested an association between IBD and RA (49–51). Other prospective studies have reported that IBD can increase the risk of RA (52, 53). A meta-analysis of the association between IBD and RA showed that IBD increased RA prevalence (54). These previous results are in line with the present MR results. Additionally, the potential genetic overlap between IBD and RA provided some genetic evidence to support these results. A US retrospective study found that CD patients were more likely to develop RA than UC (55). The findings of a meta-analysis on the risk of rheumatoid arthritis in inflammatory bowel disease have confirmed this (54). The CD and UC are commonly characterized as Th1-dominant and Th2-dominant, respectively (56, 57), while RA is often classified as a Th1-driven disease (58). Therefore, there is a closer relationship between CD and RA.In the overall genetic correlation analysis, we observed that RA was significant with CD and not with UC.A study published by Afroz et al. mentions that the expression of HLA-DOB is significantly upregulated *in vivo* in RA patients (59). The human leukocyte antigen HLA-DOB plays an important role in viral infections by influencing multiple alleles involved in antigen presentation (60, 61). In addition, a previous analysis of the colonic transcriptome found that HLA-DOB was significantly upregulated in patients with CD, but not in patients with UC (62). From the above, it is clear that HLA-DOB expression and the virus-induced autoimmunity it generates might be the mechanism for the pathogenesis of RA combined with CD. This also provides an explanation for the discordant genetic effects of RA on CD and UC observed in our study.

The gut–joint axis is emerging as a new research hotspot in the biomedical field; however, the action mechanism of the gut–joint axis is poorly understood. A recent study published in Nature Communications has reported that patients with newly-onset RA show increased serum connexin levels and decreased intestinal tight junction protein levels. In a mouse model for arthritis, the gut produced inflammatory lesions that developed earlier than did arthritis, and targeting intestinal mucosal proteins alleviated arthritis symptoms (63). The gut–joint axis is associated with the activation of the innate immune system of the host, in which gut microbe-related factors, including short-chain fatty acids, bile acids, and tryptophan and its metabolites, and intestinal flora play a crucial role (64). IBD and RA are immune-mediated diseases, and they interact with each other to form the gut–joint axis, and the overlapping genetic structure may be the potential basis for their action mechanisms.

The results of KEGG-enriched pathways is very interesting. EBV infection disrupts cellular immunity, and its presence increases IBD and RA incidences (65, 66). JAK–STAT signaling is an essential pathway in immunology, which is generally activated, phosphorylated, and dimerized by the phosphorylation of intracellular JAK, which further activates STAT. Then, the proteins migrate to the nucleus as homo/heterodimers to bind to specific DNA-binding sites and eventually participate in the immunological processes of IBD and RA (67–69). The first-generation JAK inhibitor, tofacitinib, is being considered for IBD and RA treatment (70).

Based on conjfdr and MTAG analysis, the genes that warrant attention are ubiquitin-conjugating enzyme E2 L3 (UBE2L3) and interferon regulatory factor 5 (IRF5). The former is a significant gene associated with both IBD and CD, while the latter is a significant gene unique to UC. Conjfdr suggests that they demonstrate a positive association (Z > 0) between the two diseases and serve as their susceptibility genes. UBE2L3 encodes a ubiquitin-conjugating enzyme, which regulates interferon and TLR7/9 signaling pathways (71). According to two GWAS studies, UBE4L3 was a possible new risk gene for IBD as it played an important role in intestinal pathogenesis (72, 73). Variation in the UBE2L3 region is a critical locus for altered immune mechanisms in RA (74, 75). Studies have reported a correlation between IRF5 and UC. In a clinical trial investigating thalidomide’s efficacy in treating UC, it was found that thalidomide effectively modulated M1 macrophage polarization by targeting IRF5, ultimately leading to an amelioration of inflammation (76). Yang et al. conducted immunohistochemistry, western blotting, and quantitative real-time polymerase chain reaction analyses on peripheral blood and mucosal samples from IBD patients, revealing elevated levels of IRF5 expression significantly correlated with disease activity. This suggests that IRF5 could serve as a potential therapeutic target for UC (18). Furthermore, research indicates that the association between IRF5 and UC extends beyond the European population to include the Han population (77).

The LDSC–SEG analysis indicated that the spleen was associated with both diseases. As the largest secondary lymphoid organ in the body, the spleen exhibits diverse immune functions and plays a role in hematopoiesis and erythrocyte clearance (78). A study has shown the use of spleen-targeted H2S donor-loaded liposomes for effective immunotherapy against IBD (79). Additionally, splenic sympathetic overactivity drives inflammation and disease progression in immune disorders, such as IBD and RA, via the G-coupled protein receptor kinase pathway (80).

In the present study, we used powerful statistical inference methods, such as conjFDR, to examine the directional association of all overlapping genetic variants; this examination was independent of genetic correlation and improved qualitative confidence. Nevertheless, the study has some limitations. First, our study was conducted on the European population; thus, the present conclusions might not apply to other populations. Second, the identified lead SNPs may be associated with other disease-causing SNPs, and some genetic and environmental risk factors, such as rare variants and infections, may confound the present results. Finally, the validation of an independent cohort was not performed because the present study was based on the methodological aspects of computer simulation with publicly available GWAS data.

## 5 Conclusion

In conclusion, we obtained evidence for a shared genetic architecture between IBD and RA by targeting large-scale GWAS pooled data and using diverse genomic approaches. Furthermore, we found local and overall genetic correlations and causal associations between IBD and RA by mining genetic risk loci and tissues that were common to both. The present findings provide valuable insights into the shared genetic architecture between IBD and RA, providing necessary information for devising treatment strategies for IBD and RA.

## Data availability statement

The original contributions presented in the study are included in the article/**Supplementary Material**. Further inquiries can be directed to the corresponding author.

## Author contributions

QL: Writing – original draft, Visualization. GCa: Writing – original draft, Methodology, Formal analysis, Data curation, Conceptualization. YW: Writing – original draft, Visualization. GCh: Writing – review & editing, Supervision.
