# Supplementary material for: Inflammatory bowel disease and rheumatoid arthritis share a common genetic structure
Source: Front Immunol. 2024 Jun 13;15:1359857. doi: 10.3389/fimmu.2024.1359857 (PMC11208460; doi:10.3389/fimmu.2024.1359857)

Supplementary Figure 1.HESS analysis of ukb-b-9125 and IBD, CD and UC. The top and middle sections of each subgraph represent local genetic correlations and covariances, respectively, and the colored bars represent loci with significant local genetic correlations and covariances. The bottom portion represents the local snp heritability of an individual trait, and the colored bars represent loci with significant local snp heritability. (A) Local genetic correlation between IBD and ukb-b-9125.(B) Local genetic correlation between CD and ukb-b-9125.(C) Local genetic correlation between UC and ukb-b-9125.IBD, inflammatory bowel disease;CD, Crohn's disease; UC, ulcerative colitis.


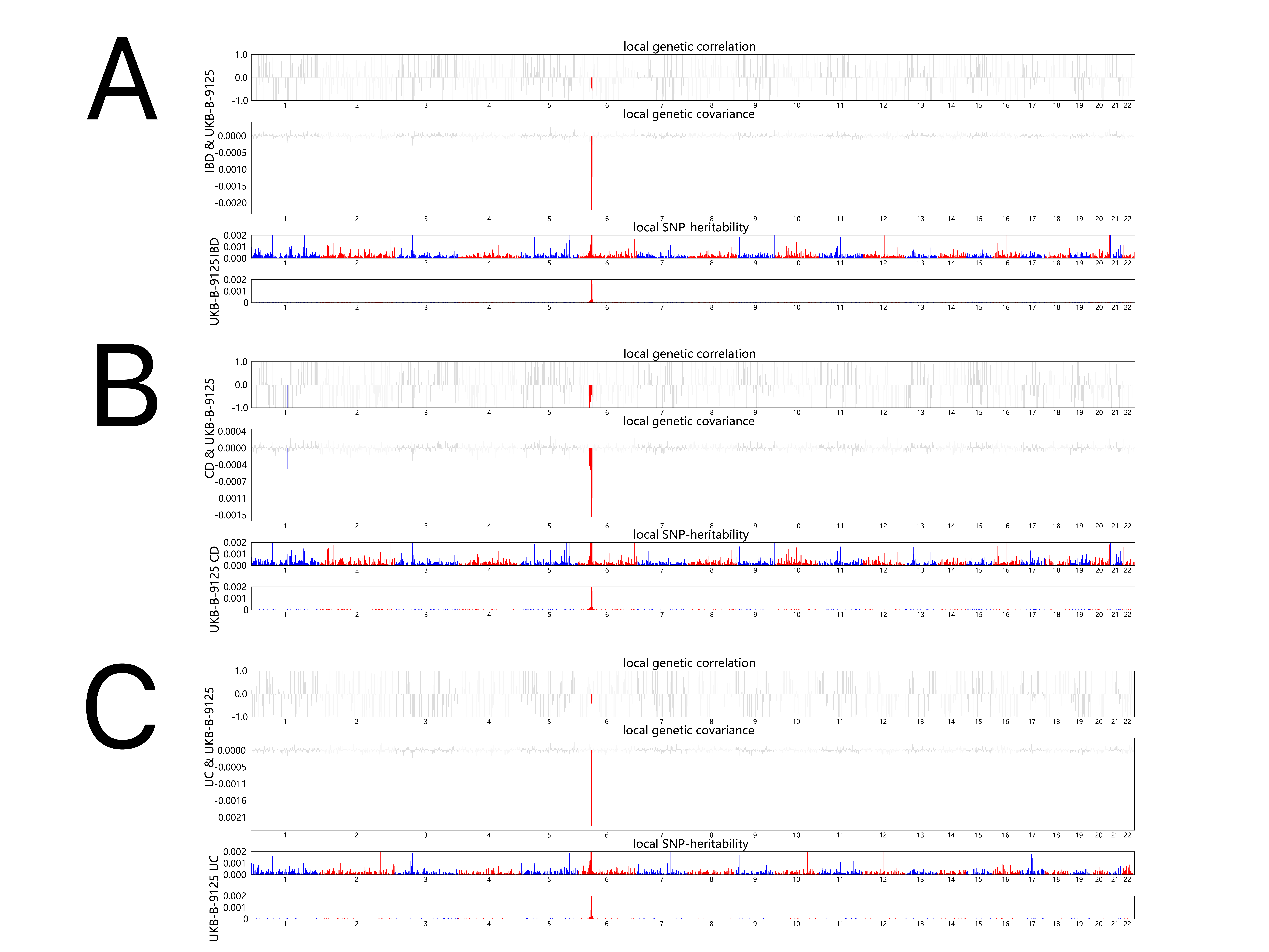


Supplementary Figure 2. Conditional quantile-quantile plot. The dashed line indicates the expected line under the null hypothesis, and the deflection to the left indicates the degree of pleiotropic enrichment.(A)IBD-ukb-b-9125.(B)ukb-b-9125-IBD.(C)CD-ukb-b-9125.(D)ukb-b-9125-CD.(E)UC-ukb-b-9125.(D)ukb-b-9125-UC.IBD, inflammatory bowel disease;CD, Crohn's disease; UC, ulcerative colitis.


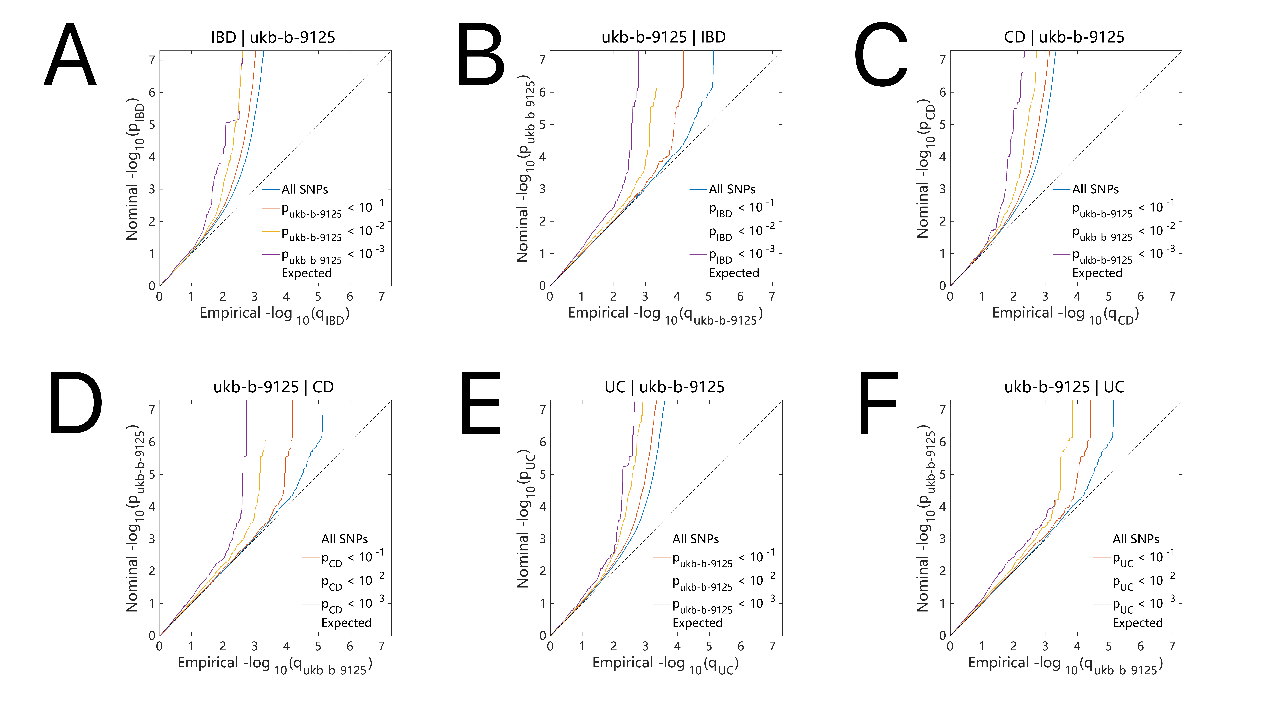


Supplementary Figure 3. (A)ConjFDR Manhattan plot of IBD and ukb-b-9125.(B)ConjFDR Manhattan plot of CD and ukb-b-9125.(B)ConjFDR Manhattan plot of UC and ukb-b-9125.The shared risk loci between ukb-b-9125 and IBD, CD and UC were marked. The statistically significant causality is defined to be conjFDR <0.01. IBD, inflammatory bowel disease;CD, Crohn's disease; UC, ulcerative colitis.


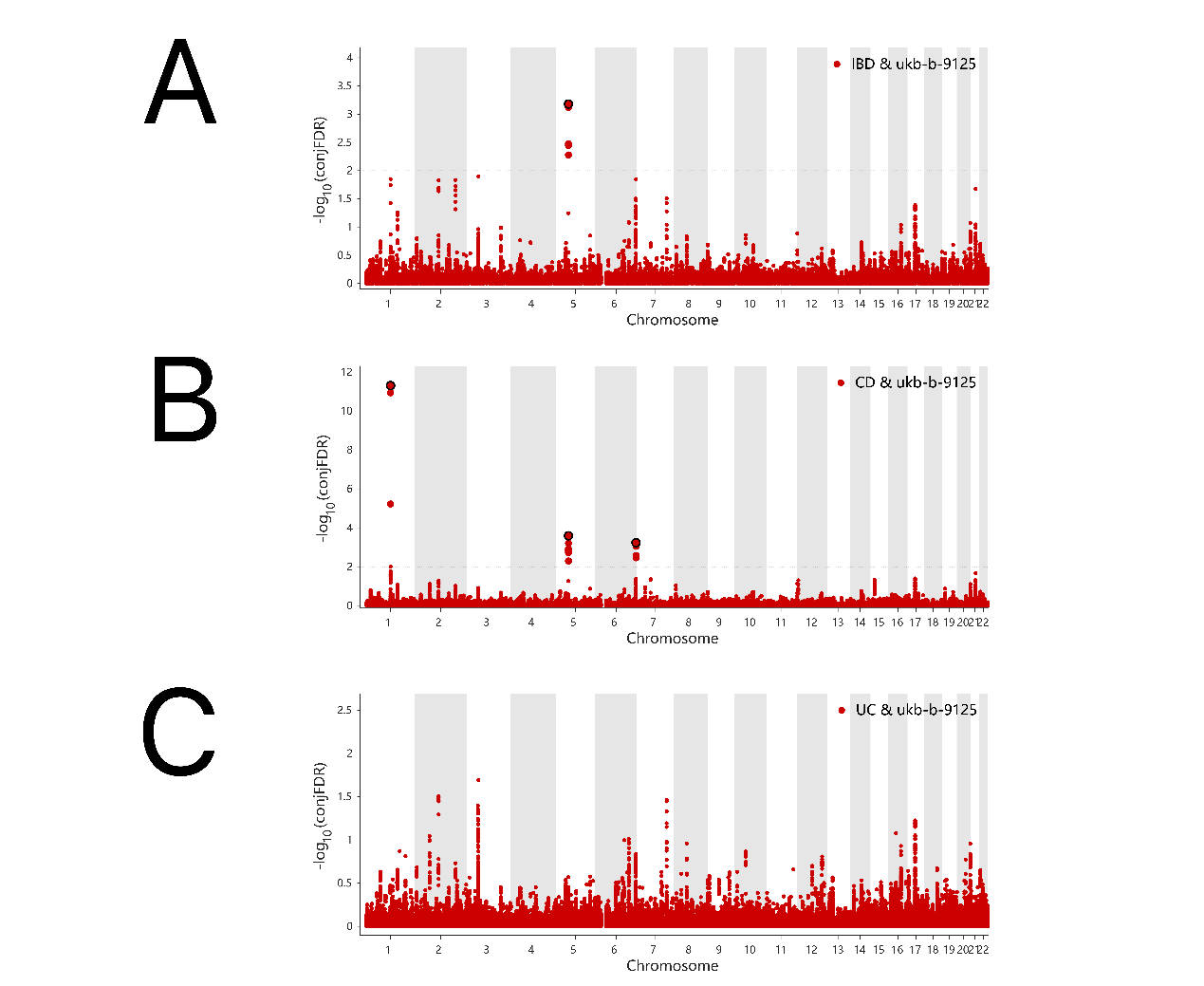


Supplementary Figure 4. The enrichment results for CD mapping genes.(A). GO enrichment analysis at biological process.(B). GO enrichment analysis at molecular function.(C). GO enrichment analysis at cell composition.(D). KEGG analysis.CD, Crohn's disease.


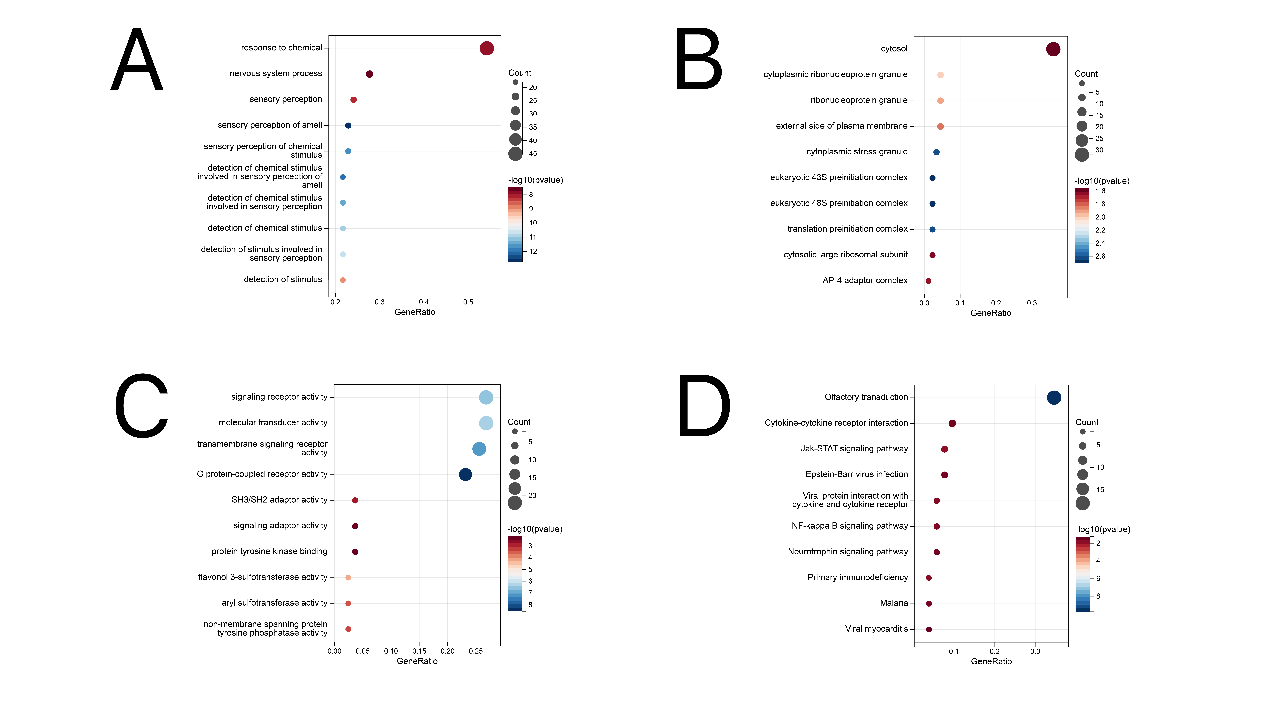


Supplementary Figure 5. The enrichment results for UC mapping genes.(A). GO enrichment analysis at biological process.(B). GO enrichment analysis at molecular function.(C). GO enrichment analysis at cell composition.(D). KEGG analysis.UC, ulcerative colitis.


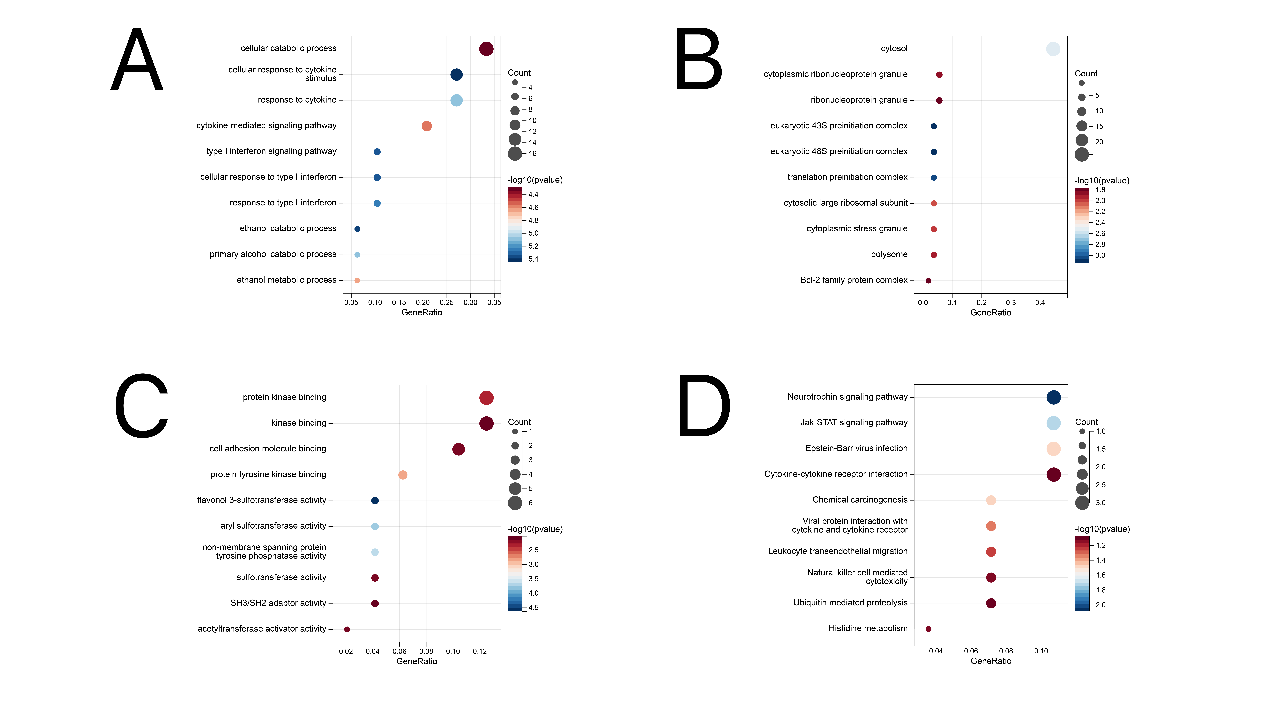


Supplementary Figure 6. The intersection of genetic risk sites identified by conjfdr and MATG analysis.(A). IBD-RA.(B). CD-RA.(C).UC-RA.RA,Rheumatoid arthritis; IBD, inflammatory bowel disease;CD, Crohn's disease; UC, ulcerative colitis.


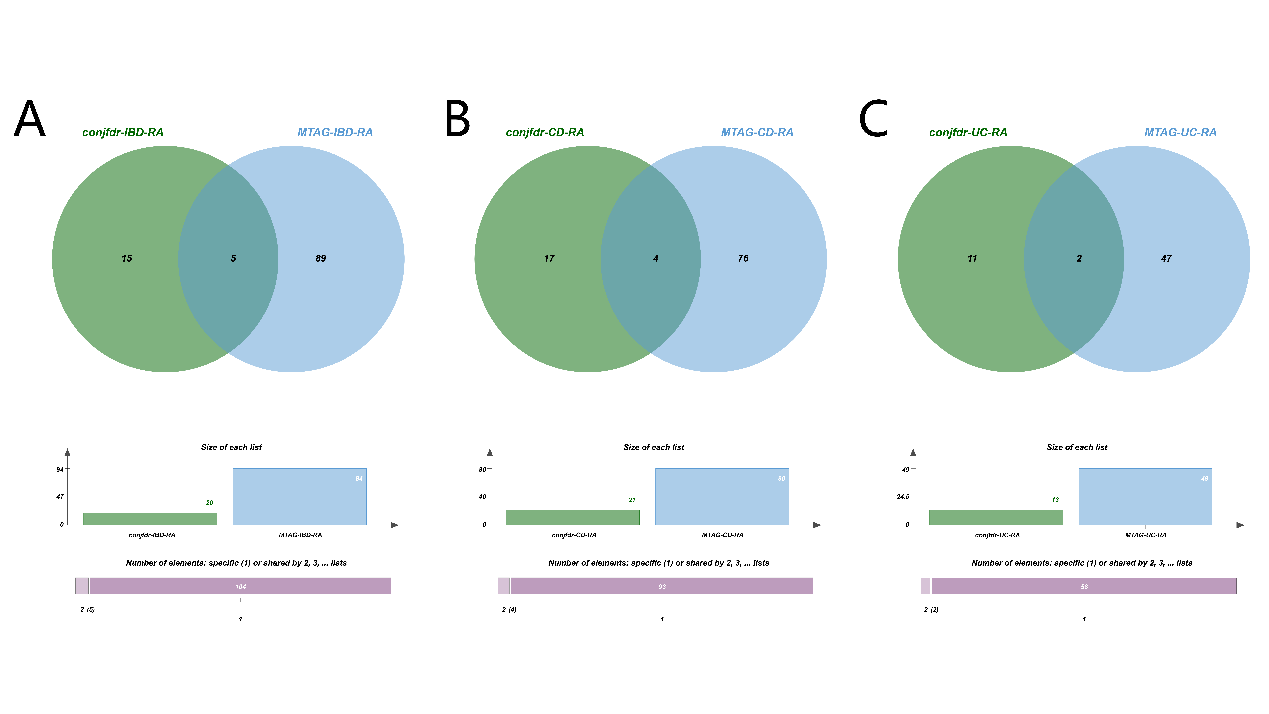

Supplement: Supplementary file 1 [file DataSheet_1.docx]
